# Eating Behaviours and Food Cravings; Influence of Age, Sex, BMI and *FTO* Genotype

**DOI:** 10.3390/nu11020377

**Published:** 2019-02-12

**Authors:** Hanan M. Abdella, Hameida O. El Farssi, David R. Broom, Dawn A. Hadden, Caroline F. Dalton

**Affiliations:** 1 Biomolecular Sciences Research Centre, Sheffield Hallam University, Sheffield S1 1WB, UK; hanan.m.abdella@student.shu.ac.uk (H.M.A.); hameida.o.elfarssi@student.shu.ac.uk (H.O.E.F.); d.hadden@shu.ac.uk (D.A.H.); 2 Academy of Sport and Physical Activity, Sheffield Hallam University, Sheffield S10 2BP, UK; d.r.broom@shu.ac.uk

**Keywords:** body mass index, cognitive restraint, eating behavior, emotional eating, food craving, uncontrolled eating, weight gain, weight loss

## Abstract

Previous studies indicate that eating behaviours and food cravings are associated with increased BMI and obesity. However, the interaction between these behaviours and other variables such as age, sex, BMI and genetics is complex. This study aimed to investigate the relationships between eating behaviours and food cravings, and to examine the influence of age, sex, body mass index (BMI) and fat mass and obesity-associated (*FTO*) genotype on these relationships. A total of 475 participants (252 female, 223 male, BMI: 25.82 ± 6.14 kg/m^2^, age: 30.65 ± 14.20 years) completed the revised 18-question version of the Three Factor Eating Questionnaire (TFEQ-R18) to assess cognitive restraint, uncontrolled eating and emotional eating, and the Food Cravings Inventory (FCI) to assess cravings for fatty food, sweet food, carbohydrates and fast food. DNA samples were genotyped for the rs9939609 polymorphism in the obesity-linked gene *FTO*. Questionnaire data was analysed for associations between the TFEQ-R18 and FCI subscales for the whole study group, and the group divided by sex, genotype and age (≤25 years versus >25 years). Finally, mediation analysis was used to explore the relationships between BMI, cognitive restraint and food cravings. *FTO* AA + AT genotype was associated with increased BMI, but not with differences in eating behavior scores or food craving scores; age was associated with increased BMI and decreases in food craving scores in which this effect was stronger in women compared to men. Increased cognitive restraint was associated with decreased food craving scores in the ≤25 years group. Mediation analysis demonstrated that in this group the association between BMI and reduced food cravings was mediated by cognitive restraint indicating that in this age group individuals use cognitive restraint to control their food cravings. The positive correlation between age and BMI confirms previous results but the findings of this study show that age, sex, *FTO* genotype and BMI have an influence on the relationships between eating behaviours and food cravings and that these variables interact.

## 1. Introduction

Obesity is a global problem; worldwide 39% of adults are classified as being overweight and 13% as having obesity [1]. It is now recognised that the development of obesity is influenced by a complex interaction between genetics and psychological factors, such as eating behaviours and food cravings.

Behaviours related to food intake which influence the frequency of eating, meal size, meal content, and attitude to meals are described as ‘eating behaviours’. Eating behaviours can influence the amount of energy consumed by an individual thus predisposing to an increased risk of obesity [2]. The most commonly studied types of eating behaviours are uncontrolled eating (UE), emotional eating (EE), and cognitive restraint (CR). UE refers to a tendency to overeat, with the feeling of being out of control. EE reflects a propensity to overeat in response to negative emotions (i.e., when feeling lonely, anxious or depressed) [3]. CR refers to a tendency to consciously restrict food intake instead of using physiological cues (i.e., hunger and satiety) as regulators of intake [4]. Previous studies have shown an association of eating behaviours with weight gain and increasing body mass index (BMI) [5,6] which are directly related to food intake [5].

Food craving is defined as a strong, irresistible desire to consume a specific type of food [7], this desire is extremely common with some studies estimating it is experienced by more than 90% of the adult population [8,9]. Food cravings can be categorised by both appetitive and aversive components and can be prompted by certain emotional conditions, such as psychological or physical stress, anxiety, depression, anger, or psychological reactance to food [10,11]. Increased food cravings have been associated with increased BMI [5,12].

Fat mass (FM) and obesity-associated *FTO* is the first obesity risk gene recognised by genome wide association studies (GWAS) and is the gene most strongly associated with an increase in BMI [13]. Single-nucleotide polymorphisms (SNPs) that cluster in the first intron of the *FTO* gene show the strongest association with BMI (∼0.35 kg/m^2^ per allele), and obesity risk [14,15]. *FTO* is highly expressed in the hypothalamus, a region involved in the regulation of food intake and energy expenditure [16,17]. Previous studies have stated that the BMI-increasing allele A of the *FTO* variant is associated with higher energy intake and higher fat and carbohydrates intake compared to TT homozygotes [18,19]

An influence of *FTO* on eating behaviours has been shown, although the results are contradictory. Habron et al., suggest that the *FTO* risk allele was associated with differences in eating behaviours in adults with overweight or obesity [20] and similar findings have been shown in normal weight controls [21]. In contrast, in a group of adolescents and children, *FTO* genotype was associated with BMI but did not influence eating behaviour [22]. The *FTO* gene has been reported to influence food cravings, with individuals carrying the obesity-susceptible A allele having higher total food cravings compared to TT homozygotes which correlates with higher BMI [23].

Sex and age also influence eating behaviours and food cravings. Previous studies highlight that females have higher scores for CR than males but report inconsistent sex effects for UE or EE [24,25,26,27]. Other studies found significant differences between sexes with females scoring higher on EE and CR but equal mean scores for UE [3,28,29]. Löffler et al. found that females scored significantly higher than males in all subscales of eating behaviours and also found individuals over 60 years old had significantly higher mean scores than people under 60 years for CR, but lower mean scores for UE and EE [30].

Regarding the effects of sex and age on food cravings, Imperatori et al. found that females with overweight or obesity were more likely to experience cravings than males [31] and Chao et al. highlights that females had significantly higher cravings for sweets than males [32]. A previous study also stated that women reported significantly more cravings for chocolate and for sweets than did men. However, craving for sweets declined with age among women [33]. These findings are in agreement with another two studies that included older females in reporting a negative association between food cravings and age, although neither of these studied controlled for BMI [34,35]. Food cravings declined with age, but this age effect differed across variants of *FTO* rs9939609: while TT homozygotes showed the typical age-related decline in food cravings, there was no such decline among A carriers, suggesting that they are at risk for increases in weight gain over the course of aging as fat mass often increases with age [23]. The decline in food cravings with age may also be related to age-dependent changes in taste sensitivity that have been reported [36,37], and may also be responsible for age-related declines in food intake.

### Aim

The aim of the current study is to investigate the interactions between eating behaviours, food cravings and BMI. Previous findings indicate that when considering the influence of eating behaviours and food cravings on obesity, it is important to take into account other variables that are known to also influence these measures including genetics, age and sex. It is also important consider which associations are mediated by other relevant variables. This study therefore (1) investigates the interactions between eating behaviours, food cravings and BMI (2) determines the influence of *FTO* genotype, sex and age on these interactions and (3) uses mediation analysis to explore the role of these mediators in the interactions.

## 2. Materials and Methods

### 2.1. Study Participants

Participants were recruited through advertisements at Sheffield Hallam University, Sheffield, UK. The participants included students at the university, staff at the university and friends and family of students at the university. Ethical clearance for the study was granted by the Ethics Committee of the Biomolecular Research Centre, Sheffield Hallam University and all volunteers provided written informed consent before taking part in the study. To be included in the study participants needed to be ≥18 years. People who were pregnant or breastfeeding, who had a history of serious psychiatric illness or were taking medication known to affect appetite or metabolism were excluded. A total of 475 participants (*n* = 252 F, *n* = 223 M), average BMI 25.82 ± 6.14 kg/m^2^, average age 30.65 ± 14.20, age range 18–81 were included in this study. 249 of the participants were aged 25 years or younger (≤25 years), with 226 participants aged over 25 years (>25 years). We were interested in studying eating behaviours at a stage in life before patterns had become fully established. We considered previous work which shows that life events such as co-habiting, marriage, securing a job and having children are associated with changes in eating patterns and weight gain [38,39,40]. We chose 25 years as the cut-off because for many in our study group up to this age would be prior to the aforementioned life events.

### 2.2. Anthropometry

Height was measured to the nearest 0.1 cm using a wall-mounted stadiometer (Seca, Hamburg, Germany). Participants stood barefoot with their heels together against a wooden back plate keeping their arms loosely by their side. The measurement was recorded when the head was in the Frankfort Plane i.e., a horizontal line between the lower orbits of the eyes and the external auditory meatus. Body mass was measured to the nearest 0.01 kg using a balance beam scale (Avery, Birmingham, UK). Participants wore light clothing, removed their shoes and jewelry and were told to remove anything from their pockets. BMI was calculated as the body mass in kilograms divided by the square of height in meters.

### 2.3. Eating Behaviours

Eating behaviours were measured using the revised 18 items Three Factor Eating Questionnaire (TFEQ-R18) [3]. This is a shortened version of the original 51-item TFEQ developed by Stunkard and Messick (1985) [41]. The questionnaire measures three different subscales of eating behaviour: (1) Cognitive restraint (CR) defined as the conscious restriction of food intake aimed to control body weight and/or to promote weight loss comprised of six items e.g., “I consciously hold back at meals in order not to gain weight”. (2) Uncontrolled eating (UE) defined as the tendency to eat more than usual due to a loss of control over intake with a subjective feeling of hunger. Comprised of nine items e.g., “When I see a real delicacy, I often get so hungry that I have to eat right away”. (3) Emotional eating (EE) defined as an inability to resist emotional cues, eating as a response to different negative emotions. Comprised of three items e.g., “When I feel blue, I often overeat”. The 18 items are measured on a 4-point response scale (definitely true: 4, mostly true: 3, mostly false: 2, definitely false: (1) and items scores are summated into subscale scores: CR, UE and EE. Previous studies have reported that TFEQ-R18 has adequate internal consistency reliability coefficients for the three subscales, as well as for the whole questionnaire (0.75–0.87) [3].

### 2.4. Food Cravings

Food cravings were measured using the Food Craving Inventory (FCI), a self-report measure that assesses general and specific food cravings [42]. The FCI is a valid and reliable measure of specific food cravings in a subjective manner within the previous month. The questionnaire consists of 28 items measuring the frequency of cravings for a specific food item on a scale of 0—never to 4—almost every day. The FCI has four subscales: (1) high fat foods (fried chicken, sausages, gravy, fried fish, bacon, steak, sausage rolls), (2) sweets (brownies, cookies, chocolate, doughnuts, cakes ice-cream, sweets, sponge cake), (3) carbohydrates (breads, pancakes, biscuits, sandwich bread rice, baked potato, pasta, cereal, danish pastry, crisps) and (4) fast food (hamburger, French fries, pizza). A total score for each subscale is calculated by the summation of each food item score for each participant. The FCI has demonstrated acceptable internal consistency reliability and test-retest reliability in adults [42]. The inventory shows adequate internal consistency (Cronbach’s a of 0.86 for the fats and sweets subscales, 0.84 for the starches/complex carbohydrates subscale, 0.76 for the fast-food subscale, and 0.93 for the inventory as a whole [42]. Further psychometric support for the FCI has been established in diverse community and clinical samples [43,44].

### 2.5. Genotyping

Buccal samples for genotyping were collected using Easicollect devices (GE Healthcare Life Sciences, Little Chalfont, UK). DNA was extracted from the FTA card contained in the device using a QIAamp DNA mini kit (QIAGEN, Manchester, UK) according to the manufacturer’s instructions. Genotyping was performed using Taqman SNP genotyping assays (Applied Biosystems, Life Technologies, Ltd., Paisley, UK). *FTO* gene, SNP ID: rs9939609 was investigated in this study.

### 2.6. Statistical Analysis

**Figure 1:**
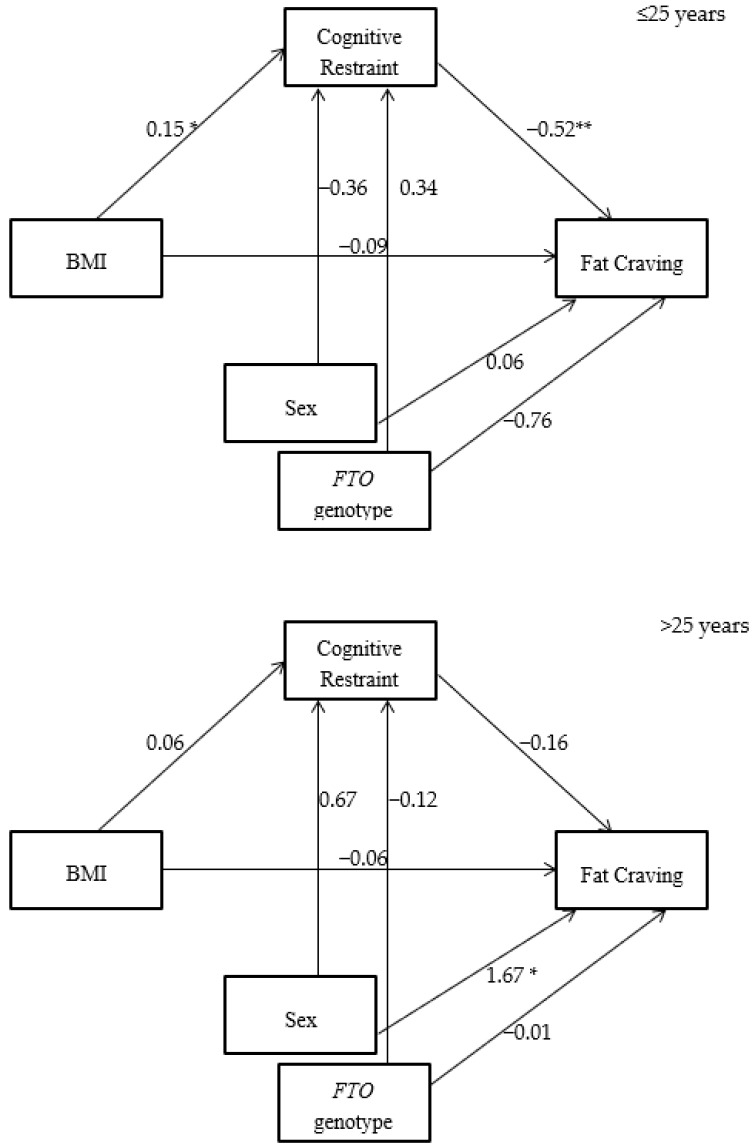
CR mediates the relationship between body mass index (BMI) and cravings for fatty food in the 25 years group but not in the >25 years group (* *p* < 0.05; ** *p* < 0.01).

**Figure 2:**
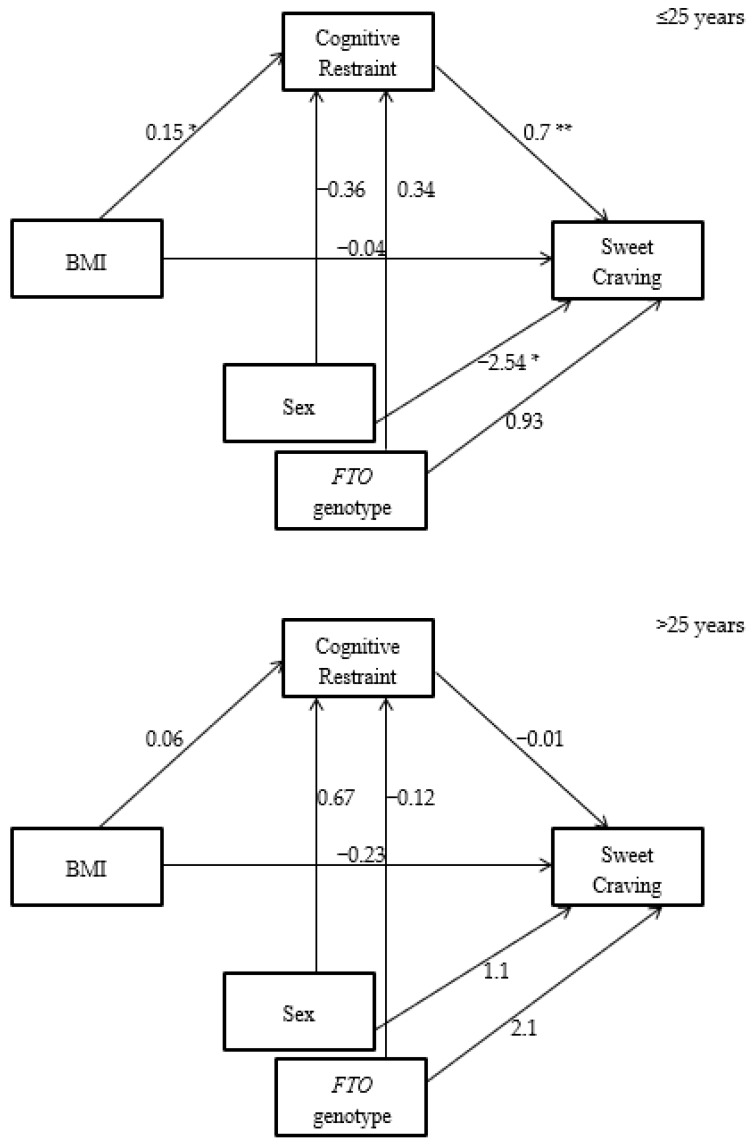
CR mediates the relationship between BMI and cravings for sweet food in the ≤25 years group but not in the >25 years group (* *p* < 0.05; ** *p* < 0.01).

Statistical Package for the Social Sciences (SPSS software version 24, IBM) was used for the statistical analysis with significance accepted if *p* < 0.05. Distribution of data was checked using the Kolmogorov–Smirnov Test and descriptive statistics were calculated. Pearson correlation was undertaken to explore association between variables including age, BMI, eating behaviours and food craving. *T*-tests were performed to assess gender and genotype differences in age, BMI, eating behaviours and food cravings. To explore relationships between variables mediation analysis with bootstrapping as recommended by Preacher and Hayes (2008) [45] was used; the SPSS macro provided by Hypotheses were tested in SPSS using PROCESS Model 59 [46], with using model 4,10,000 bootstrapped estimates and a 95 percent confidence interval. This model estimates a moderated indirect effect by evaluating the impact of the moderator on the indirect effect, as well as the predictive relationship to the direct effect. For the mediation model (Figure 1 and Figure 2), BMI was the independent variables, eating behaviours was the mediator variable, and food craving was the dependent variable. Age, sex and *FTO* genotype were the covariates. For outcome data, effect sizes were calculated using Cohens *d*. Thresholds were set at 0.0–0.19 for a trivial effect, 0.2–0.49 for a small effect, 0.5–0.79 for a medium effect and ≥0.8 for a large effect [47,48].

## 3. Results

### Descriptive Statistics

Descriptive statistics for the primary variables for the overall sample, by sex, genotype and age are shown in Table 1 and Table 2. There were no significant differences in mean BMI between men and women but there was a significant difference between carriers of the *FTO* TT genotype compared to those with AT + AA genotype (24.62 ± 5.29 versus 26.52 ± 6.55 kg/m^2^, *p* = 0.001). Table 1 also shows the results of mean food craving scores; women had higher mean scores for carbohydrate cravings compared to men (14.73 ± 5.99 versus 13.59 ± 5.74, *p* = 0.036). There were no differences in eating behaviour scores between men and women and no difference in scores for food cravings or eating behaviours between the different genotypes. BMI was higher in the >25 years group, but craving for fat and carbohydrate were higher in the ≤25 years group, as were scores for uncontrolled eating and emotional eating.

Table 3 shows that there is a positive relationship between age and BMI (*r* = 0.509 **) and an inverse relationship between age and fatty food cravings (*r* = −0.107 *), UE (*r* = −0.222 **) and EE (*r* = −0.184 *) for the whole sample. There is an inverse relationship between BMI and fatty food cravings (*r* = −0.180 **), and a positive relationship between BMI and CR (*r* = 0.128 **).

Table 4 shows that there is an inverse relationship between CR and fatty food cravings (*r* = −0.259 **), sweet food cravings (*r* = −0.138 **) and fast food cravings (*r* = −0.167 **). There is a positive relationship between UE and carbohydrate cravings (*r* = 0.164 **) and there is an inverse relationship between EE and fatty food cravings (*r* = −0.197 **), and fast food cravings (*r* = −0.125 **).

Table 5, Table 6 and Table 7 show the data dichotomised by sex (male and female), genotype (TT genotype and AA + AT genotype), and age (≤25 years and >25 years).

When exploring the differences between men and women, Table 5 shows that in women there is an inverse relationship between age and fat cravings (*r* = −0.157 *) and carbohydrate cravings (*r* = −0.195 **), in contrast to men where these relationships are not seen. In men there is an inverse relationship between BMI and CR (*r* = 0.210 **) in contrast to women where there is no relationship. In men there were also inverse relationships between CR, EE and fast food cravings which were not seen in women.

Table 6 shows that *FTO* genotype significantly affects the relationships between age, BMI, eating behaviours and food cravings. The decline in emotional eating with age was greater in the AA + AT genotype group; in addition the inverse relationship between BMI and fat craving was stronger in this group compared to the TT group.

As there was such a strong effect of age on eating behaviours and food cravings, we investigated the participant group split by age. The results show differences between the ≤25 years and >25 years age groups. There is an inverse relationship between sweet cravings and EE in the younger group, whereas in the older group this relationship is positive. A similar difference is seen for the relationship between fat cravings and EE. In the younger group there are relationships between fast food cravings and CR and EE which are not present in the older group.

## 4. Discussion

The aim of this study was to explore the relationships between eating behaviours and food cravings, and examine the influence of sex, BMI, age and *FTO* genotype on these relationships. We used the TFEQ-R18, FCI, and genotyped for the rs9939609 *FTO* polymorphism to study 475 individuals. We analysed data from this group split by sex, *FTO* genotype and age (≤25 years versus >25 years old). We then used mediation analysis to investigate possible mechanisms underlying the association between some of the relationships observed. The main findings are as follows.

Mean scores for the three subscales of the TFEQ-R18 and the four subscales of the FCI were similar to values previously reported. There were no differences between mean scores for BMI, eating behaviours or food cravings split by sex, with only cravings for carbohydrate being higher in women. This agrees with previous studies [28,49], but is in contrast with others which found higher scores for eating behavior subscales in women [30,50]. This difference may be due to the age of the groups studied; the average age of our group was 31 years, whereas the average age of the participants in these two studies was 50 and 48 years respectively indicating that sex differences in eating behaviours may become more apparent with increasing age. When the group was analysed by genotype the AA + AT group had a mean BMI of 26.52 kg/m^2^ compared to the TT genotype group who had a mean BMI of 24.62 kg/m^2^, this is consistent with the findings of previous studies which also found that carriers of the risk allele A had higher BMI scores compared to people with the TT genotype [13,51]. We did not find differences in TFEQ-R18 or FCI scores when the groups were split by genotype, which is in agreement with a recent study of children by Rivas et al. [52], which also did not find differences in subscale scores for the TFEQ-R18. This is in contrast to other findings which describe higher food cravings associated with the A allele in a group with an average age of 50 years [23]. These differences agree with our findings presented in Table 6 which demonstrate an influence of *FTO* genotype on age-related eating behaviours.

We report highly significant relationships between eating behaviours and food cravings, with BMI increasing with age, whilst cravings for fatty food decreased, and UE and EE decreased with age, in agreement with earlier studies [33,34,35]. The data presented in Table 2 also confirms this; when the two age groups were compared the scores for cravings for carbohydrates and fat, and the scores for emotional eating and uncontrolled eating were all higher in the ≤25 years group compared to the >25 years group. This may reflect changes in eating patterns associated with stages of life; with younger people tending to consume less healthy food [53,54], it may also be due in part to age-related changes in taste sensitivity [36,37]. We also found that increased BMI was associated with a decrease in cravings for fatty foods but an increase in cognitive restraint, confirming previous results by Johnson et al. who also proposed that high cognitive restraint in normal weight individuals increases the risk of overeating tendencies when restraint is relaxed, thus leading to further increases in BMI [55]. We found highly significant relationships between eating behaviours and food cravings; increased cognitive restraint correlated with lower cravings for fatty foods, sweet foods and fast foods, increased uncontrolled eating was associated with increased cravings for carbohydrates, and increased emotional eating was associated with lower cravings for fatty foods and fast foods. This is consistent with previous findings; decreased food cravings are associated with increased fMRI-FCR of brain regions that regulate executive control over ingestion [56] and with cognitive reappraisal strategies, in particular those focusing on the benefits of not eating unhealthy foods, this could potentially increase the ability of individuals with obesity to inhibit appetitive motivation and reduce unhealthy food intake [57].

When the group was split by sex analysis showed that the relationships between age and decreasing food cravings were stronger in women compared to men. However, the relationships between eating behaviours and food cravings were not affected by sex. *FTO* genotype influenced some of the relationships between BMI, age, eating behaviours and food cravings; in particular the inverse relationship between BMI and fatty food craving was stronger in the AA + AT genotype group compared to the TT group. We also saw an effect of the *FTO* genotype on the relationship between age and emotional eating, with age-related decline in this behavior only present in the AA + AT genotype group.

We split the group into a younger and older age group based on assumptions of life changes that could induce eating behaviours. We observed clear differences between the ≤25 years group compared to the >25 years group. The inverse relationships between CR and EE with food cravings for fatty, sweet and fast foods were much stronger in the ≤25 years group compared to the >25 years group. In the case of the relationship between sweet food cravings and emotional eating there was a negative correlation in the ≤25 years group, but in the >25 years group there was a significant positive correlation. There was also a strong relationship between age and BMI in the >25 years group but not in the ≤25 years group. 

We used mediation analysis to investigate these relationships. This showed that in the ≤25 years group for both cravings for fatty food and cravings for sweet food CR mediated almost all of the relationships with BMI, demonstrating that it is very likely that the people in this group with higher BMI are using CR to suppress food cravings. This was in contrast to the >25 years group where this pattern was not seen. In the ≤25 years group sweet cravings were strongly influenced by sex, with a more pronounced inverse relationship between BMI and sweet cravings seen in women. When CR was first investigated as an eating behavior the strong correlation observed between CR and BMI led to the conclusion that CR was a type of unhealthy eating behavior [4], and that people who restrained were more susceptible to binge eating, thereby leading to increased BMI. Thus, CR as an eating behavior was seen to drive an increase in BMI. However, many early studies were carried out in participants with overweight or obesity and in older demographic groups; in these groups CR may indeed be driving an increase in BMI. In contrast the data presented here supports that in younger age groups BMI may drive CR rather than the other way round, a suggestion which has also been made in other papers [29,57]. We have observed the same association between CR and BMI seen in previous studies, but in the ≤25 years group this was particularly associated with a decrease in food cravings. We therefore propose that in people ≤25 years, who are perhaps more self-conscious about their weight, CR is used to suppress food cravings, particularly in people with a raised BMI, hence the association between BMI and CR. This may instill habitual eating behaviours, which in later life increases the susceptibility to the cycles of restraint and binge-eating associated in some older populations with increased BMI [58]. Evidence to support this theory is provided by Rocks et al. who studied undergraduate students and found that this group, with a similar age and background to our ≤25 years group, demonstrated high dissatisfaction with body weight and high rates of disordered eating behaviours [59]. An additional possibility is that BMI also mediates the effect of age on eating behaviours and food cravings.

Our study has limitations; although many of the participants were drawn from the same geographical area there may be demographic differences between the ≤25 years group who were mostly undergraduate students, and the >25 years group who were a mixture of staff, students and people from outside the university. In addition the lifestyle and eating habits of the ≤25 years group in our study may not be fully representative of the general population in this age category. As some of the participants were friends and family of the students at the university they may share similar tastes, food preferences and cultural background which may also have influenced the results.

We did not collect detailed data on socio-economic status, education, marriage status or ethnicity in the current study so in future studies these potential confounders will be considered. The division into two age groups with a cut-off of 25 years was based on previous work that shows that life events such co-habiting, employment and having children are associated with changes in eating behaviour [38,39,40]; in our study group the age of 25 reflects a point after which these life events start to have an effect, in future studies collection of data on these variables will allow for their inclusion in the analysis. We chose to examine the effect of *FTO* genotype as this has been reported to be the most influential gene on BMI, in the future investigating a wider range of target genes would potentially reveal more information about the influence of genetics on the variables studied. A limitation of the use of self-reported questionnaires is that participants may not always be honest when answering, and the degree to which this is an issue may vary with weight status. In future studies we plan to standardise the conditions under which the questionnaires were completed to minimise this effect, and to incorporate food frequency questionnaires and DEXA analysis into our study protocol. 

## 5. Conclusions

In conclusion whilst the findings regarding the strong association between increasing BMI and age confirm previous reports the mediation analysis shows that sex, *FTO* genotype and BMI have an influence on the relationships between eating behaviours and food cravings and that these variables interact with notable differences between young and older age groups. Examining these interactions along with further polymorphisms linked to obesity could help identify those at risk of increasing BMI.

## Figures and Tables

**Table 1:** Descriptive statistics for primary variables for the overall sample by sex.

	Overall (*n* = 475)		Women (*n* = 252)		Men (*n* = 223)				
	*M*	*SD*	*M*	*SD*	*M*	*SD*	*t*	*p*	*d*
Age (years)	30.65	14.20	30.35	13.86	30.97	14.62	−0.462	0.644	0.044
Body mass index (BMI) (kg/m^2^)	25.82	6.14	25.73	6.72	25.89	5.40	−0.285	0.776	0.026
Fat craving	10.04	5.4	9.73	5.72	10.36	4.91	−1.283	0.200	0.118
Sweet craving	18.37	7.8	18.84	9.15	17.72	10.29	1.244	0.214	0.115
Carbohydrate craving	14.21	5.89	14.73	5.99	13.59	5.74	2.099	0.036 *	0.194
Fast Food craving	9.57	5.1	9.47	5.06	9.64	5.06	−0.359	0.720	0.033
Total food craving	52.19	20.4	52.78	20.31	51.32	20.35	0.779	0.436	0.071
Cognitive restraint	12.85	3.5	12.83	3.51	12.89	3.46	−0.222	0.825	0.017
Uncontrolled eating	20.75	5.4	20.82	5.35	20.62	5.63	0.405	0.686	0.036
Emotional eating	6.68	3.1	6.87	3.11	6.45	3.07	1.487	0.138	0.135

All significance tests were two-tailed. *M* = mean score; *SD* = standard deviation; *t* = *t*-test; *p* = *p*-value; *d* = Cohen’s *d*. * Denotes a significant difference between women and men.

**Table 2:** Descriptive statistics for primary variables for the overall sample, by genotype and by age.

	TT Genotype (*n* = 153)		AT + AA Genotype (*n* = 322)					≤25 Years (*n* = 249)		>25 Years (*n* = 226)				
	*M*	*SD*	*M*	*SD*	*t*	*p*	*d*	*M*	*SD*	*M*	*SD*	*t*	*p*	*d*
Age (years)	29.19	12.94	31.05	14.46	−1.29	0.212	0.135	21.41	1.58	43.48	13.94	−21.962	0.000 *	2.224
BMI (kg/m^2^)	24.62	5.29	26.52	6.55	−3.26	0.001 *	0.319	23.46	3.81	29.26	7.19	−10.093	0.000*	1.008
Fat craving	10.71	5.33	9.75	5.28	1.827	0.068	0.180	10.61	5.56	8.87	4.64	3.647	0.000 *	0.339
Sweet craving	18.16	9.10	18.86	10.04	−0.66	0.525	0.073	18.10	9.42	18.48	10.31	−0.410	0.682	0.038
Carbohydrate craving	14.55	6.194	14.33	5.61	0.388	0.698	0.037	14.52	5.91	13.39	5.69	2.068	0.039 *	0.195
Fast food craving	9.31	4.48	9.82	5.32	−1.00	0.313	0.103	9.28	5.03	9.91	5.20	−1.314	0.189	0.123
Total food craving	52.73	18.78	52.71	20.72	0.050	0.960	0.001	52.51	20.37	50.65	20.35	0.968	0.334	0.091
Cognitive restraint	12.79	3.29	13.06	3.557	−0.74	0.445	0.078	13.0	3.62	12.75	3.25	0.773	0.440	0.070
Uncontrolled eating	20.31	5.59	20.98	5.387	−1.28	0.198	0.122	21.38	5.32	19.72	5.55	3.265	0.001 *	0.310
Emotional eating	6.71	3.05	6.7	3.07	−0.300	0.764	0.003	6.97	3.07	6.25	3.03	2.527	0.012 *	0.236

All significance tests were two-tailed. *M* = mean score; *SD* = standard deviation; *t* = *t*-test; *p* = *p*-value; *d* = Cohen’s *d*. * Denotes a significant difference between genotype or age.

**Table 3:** Correlation between age, BMI, eating behaviours and food cravings in the overall sample.

	BMI	Fatty Food Craving	Sweet Craving	Carbohydrate Craving	Fast Food Craving	Cognitive Restraint	Uncontrolled Eating	Emotional Eating
Age (years)	0.509 **	−0.110 *	−0.041	−0.071	−0.037	−0.024	−0.222 **	−0.184 **
BMI (kg/m^2^)		−0.180 **	−0.075	−0.078	0.026	0.128 **	0.014	0.082

Correlations of primary variables for overall population. All significance tests were two-tailed (* *p* < 0.05; ** *p* < 0.01).

**Table 4:** Correlations between eating behaviours and food cravings in the overall sample.

	Fatty Food Craving	Sweet Food Craving	Carbohydrate Craving	Fast Food Craving
Cognitive restraint	−0.259 **	−0.138 **	0.005	−0.167 **
Uncontrolled eating	0.000	0.059	0.164 **	0.004
Emotional eating	−0.197 **	−0.029	0.043	−0.125 **

Correlations of primary variables for overall population. All significance tests were two-tailed (** *p* < 0.01).

**Table 5:** Correlation of Primary Variables by Sex.

	Women	Age	BMI	Fat Craving	Sweet Craving	Carbohydrate Craving	Fast Food Craving	Cognitive Restraint	Uncontrolled Eating	Emotional Eating
Men										
Age			0.544 **	−0.157 *	−0.107	−0.195 **	−0.117	−0.073	−0.233 **	−0.140 *
BMI		0.473 **		−0.186 **	−0.102	−0.122	0.000	0.081	−0.028	0.122
Fat craving		−0.050	−0.185 **		0.494 **	0.533 **	0.525 **	−0.225 **	−0.097	−0.241 **
Sweet craving		0.020	−0.063	0.357 **		0.330 **	0.664 **	−0.121	0.020	−0.028
Carbohydrate craving		0.071	−0.016	0.418 **	0.430 **		0.331 **	0.011	0.183 **	0.038
Fast food craving		0.045	0.050	0.435 **	0.686 **	0.379 **		−0.117	−0.049	−0.083
Cognitive restraint		0.032	0.210 **	−0.301 **	−0.139 *	0.003	−0.156 *		0.303 **	0.400 **
Uncontrolled eating		−0.215 **	0.059	0.113	0.078	0.140 *	0.047	0.173 **		0.499 **
Emotional eating		−0.232 **	0.020	−0.144 *	−0.055	0.033	−0.184 **	0.376 **	0.464 **	

Correlations of primary variables among women *n* = 252 presented above the diagonal, correlations of primary variables among men *n* = 223 are presented below the diagonal. All significance tests were two-tailed (* *p* < 0.05; ** *p* < 0.01).

**Table 6:** Correlation of primary variable by genotype.

	TT	Age	BMI	Fat Craving	Sweet Craving	Carbohydrate Craving	Fast Food Craving	Cognitive Restraint	Uncontrolled Eating	Emotional Eating
AA + AT										
Age			0.412 **	−0.082	0.014	−0.089	−0.021	−0.034	−0.215 *	−0.144
BMI		0.548 *		−0.078	−0.032	−0.077	0.076	0.154	−0.038	0.145
Fat craving		−0.103	−0.206 **		0.326 **	0.497 **	0.449 **	−0.283 **	0.020	−0.154
Sweet craving		−0.035	−0.098	0.465 **		0.280 **	0.599 **	−0.308 **	−0.068	−0.075
Carbohydrate craving		−0.042	−0.083	0.451 **	0.381 **		0.304 **	−0.080	0.135	0.024
Fast food craving		−0.024	0.016	0.501 **	0.690 **	0.333 **		−0.219 **	0.063	−0.108
Cognitive restraint		−0.020	0.101	−0.278 **	−0.114 *	0.001	−0.133 *		0.233 **	0.445 **
Uncontrolled eating		−0.220 **	0.029	−0.046	0.065	0.147 **	−0.075	0.255 **		0.442 **
Emotional eating		−0.201 **	0.053	−0.223 **	−0.050	0.011	−0.161 **	0.354 **	0.504 **	

Correlations of primary variables among TT genotype *n* = 153 presented above the diagonal, correlations of primary variables among AA + AT genotype *n* = 322 are presented below the diagonal. All significance tests were two-tailed (* *p* < 0.05; ** *p* < 0.01).

**Table 7:** Correlation of primary variable by age.

	≤25 Years	Age	BMI	Fat Craving	Sweet Craving	Carbohydrate Craving	Fast Food Craving	Cognitive Restraint	Uncontrolled Eating	Emotional Eating
>25 Years										
Age			0.009	−0.050	−0.094	0.024	−0.095	0.032	0.103	0.038
BMI		0.322 **		−0.119	−0.058	−0.095	0.046	0.174 **	0.022	0.185 **
Fat craving		0.059	−0.111		0.490 **	0.430 **	0.513 **	−0.326 **	−0.047	−0.314 **
Sweet craving		−116	−0.127	0.365 **		0.371 **	0.710 **	−0.227 **	−0.004	−0.176 **
Carbohydrate craving		0.002	0.012	0.489 **	0.397 **		0.300 **	−0.032	0.133 *	−0.045
Fast food craving		−0.186 **	−0.045	0.512 **	0.646 **	0.428 **		−0.262 **	−0.059	−0.242 **
Cognitive restraint		0.005	0.127	−0.105	−0.003	0.102	0.044		0.264 **	0.474 **
Uncontrolled eating		−0.272 **	0.143	−0.049	0.144 *	0.138	0.086	0.211 **		0.405 **
Emotional eating		−0.239 **	0.127	−0.071	0.159 *	0.127	0.032	0.217 **	0.565 **	

Correlations of primary variables among people ≤25 years *n* = 249 presented above the diagonal, and correlations of primary variables among people >25 years *n* = 226 are presented below the diagonal. All significance tests were two-tailed (* *p* < 0.05; ** *p* < 0.01).
